# Nasal carriage of methicillin-resistant *Staphylococcus aureus* (MRSA) among undocumented migrants and uninsured legal residents in Amsterdam, the Netherlands: a cross-sectional study

**DOI:** 10.1186/s13756-020-00785-8

**Published:** 2020-07-29

**Authors:** E. van Dulm, S. Klok, A. Boyd, I. K. Joore, M. Prins, A. P. van Dam, G. A. Tramper-Stranders, Y. T. H. P. van Duijnhoven

**Affiliations:** 1 Department of Infectious Diseases, Public Health Service Amsterdam, Nieuwe Achtergracht 100, 1018WT Amsterdam, the Netherlands; 2 NGO health care clinic Kruispost, Amsterdam, the Netherlands; 3 HIV Monitoring Foundation, Amsterdam, The Netherlands; 4 Department of Infectious Diseases, Public Health Service Flevoland, Lelystad, the Netherlands; 5 Division of Infectious Diseases, and Amsterdam Institute for Infection and Immunity (AI&II), Amsterdam UMC, University of Amsterdam, Department of Internal Medicine, Amsterdam, the Netherlands; 6 Department of Medical Microbiology, Amsterdam UMC, University of Amsterdam, Amsterdam, the Netherlands

**Keywords:** MRSA, Nasal carriage, Undocumented migrants, Uninsured legal residents

## Abstract

**Background:**

Nasal carriage of methicillin-resistant *Staphylococcus aureus* (MRSA) is associated with an increased risk of infection. Colonization with MRSA is observed in < 1% of the general Dutch population. Increased risk for MRSA carriage is known to occur in several key groups, one of which is asylum seekers. However, little is known about MRSA carriage among undocumented migrants and uninsured legal residents. This study aimed to determine the prevalence of nasal MRSA carriage among these groups in Amsterdam, the Netherlands.

**Methods:**

In this cross-sectional study, between October 2018 and October 2019, undocumented migrants and uninsured legal residents aged 18 years or older who were able to understand one of the study languages were recruited at an NGO health care facility in Amsterdam, the Netherlands, for general practitioner (GP) consultations. Participants were asked questions on demographics, migration history, antibiotic use and other possible risk factors for MRSA carriage and were screened for nasal MRSA carriage by selective culturing e-swabs. Characteristics of MRSA-negative and MRSA-positive participants were compared using univariable logistic regression analysis with Firth’s correction.

**Results:**

Of the 3822 eligible patients, 760 were screened for nasal MRSA carriage (19.9%). Of the 760 participants, over half were male (58%; 442/760) and originated mainly from Africa (35%; 267/760), Asia (30%; 229/760) and North or South America (30%; 227/760). In total, 705/760 participants (93%) were undocumented migrants and 55/760 (7%) were uninsured legal residents of Amsterdam. The overall prevalence of nasal MRSA carriage was 2.0% (15/760) (95%CI 1.1 to 3.2%), with no difference between undocumented migrants (14/705) (2.0, 95%CI 1.1 to 3.3%) and uninsured legal residents (1/55) (1.8, 95%CI 0.1 to 9.7%). Genotyping showed no clustering of the 15 isolates. MRSA carriage was not associated with sociodemographic, migration history or other possible risk factors. Nevertheless, this study had limited power to detect significant determinants. Three participants (3/15; 20%) harbored Panton-Valentine leukocidin (PVL)-positive isolates.

**Conclusion:**

Even though our study population of undocumented migrants and uninsured legal residents had a higher prevalence of nasal MRSA carriage compared to the general Dutch population, the prevalence was relatively low compared to acknowledged other high-risk groups.

## Background

*Staphylococcus aureus* is a commensal bacterium, but frequently causes clinically important nosocomial or community-acquired infections [1]. During infection with methicillin-resistant *S. aureus* (MRSA), treatment options are limited. Even though the number of MRSA infections is still relatively low in many Western and Northern European countries, approximately 150,000 infections of MRSA occur each year in the European Union, accounting for an estimated 7000 deaths annually [2].

Nasal carriage of *S. aureus* in general is associated with an increased risk of MRSA infection [3, 4]. Consequently, the Netherlands has taken an aggressive approach to prevent the spread of MRSA in those with MRSA carriage. As part of the Dutch MRSA search and destroy policy, all known MRSA carriers, as well as their household and in-hospital contacts, and all patients who have been admitted to foreign hospitals for more than 24 h in the previous 2 m are isolated at hospital admission [5, 6]. Isolation is prolonged until screening cultures for MRSA are negative or MRSA carriage is eradicated. Coupled with the general reluctance of prescribing antibiotics among Dutch physicians [7], the prevalence of MRSA carriage is < 0.2% of new hospital admissions in the Netherlands [8, 9].

MRSA carriage is observed in < 1% of the general Dutch population [5], yet certain groups are known to have a higher prevalence. For instance, a recent study from the Netherlands has shown that 10% of asylum seekers were carriers of MRSA [10]. This prevalence falls in line with a recent systematic review and meta-analysis among migrants in Europe in which a pooled 8% prevalence of MRSA carriage was estimated [11]. Nevertheless, other conducted studies in similar settings have reported widely varying prevalences, with a Norwegian study reporting MRSA carriage in 0.74% of asylum seekers [12] and one Finnish study reporting MRSA carriage in 21% of asylum seekers and refugees [13]. Part of this variation could be due to the variation in the countries of origin of migrants included in these studies.

Undocumented migrants (including rejected asylum seekers, migrants with expired visa and ‘directly undocumented migrants’, i.e. those who bypassed the asylum procedure) and uninsured legal residents are thought to represent a considerable fraction of migrants residing in the Netherlands, yet the exact proportion is unknown. Multiple international studies on immigration and the impact of immigration policies have shown an association between undocumented migration and poorer health outcomes [14–16]. In particular, the prevalence of infectious diseases (e.g. human immunodeficiency virus (HIV), hepatitis B virus (HBV), hepatitis C virus (HCV) and tuberculosis (TB)) tends to be higher among homeless individuals and undocumented migrants [17–21]. However, little is known about the proportion of MRSA-carriers among undocumented migrants and uninsured legal residents in the Netherlands. These individuals are known to live in more difficult socioeconomic situations (e.g. crowded living conditions) [22, 23], which could make them more vulnerable to inadequate care and possibly at higher risk for MRSA carriage. To the best of our knowledge, the prevalence of MRSA carriage in this population has not yet been studied. This study aimed to determine the prevalence of nasal MRSA carriage among undocumented migrants and uninsured legal residents in Amsterdam, the Netherlands.

## Methods

### Study design, setting and population

A cross-sectional study was designed to evaluate the prevalence of HBV, HCV, HIV and MRSA carriage in individuals seeking care at Kruispost, a low-threshold care facility for undocumented migrants and (Dutch) homeless and uninsured individuals. A sample of 1000 participants was intended to be recruited from patients visiting Kruispost for an appointment with a general practitioner (GP) during a one-year period. We based sample size on the capacity of Kruispost to recruit participants in a 12-month time span (i.e. convenience sample). Between October 2018 and October 2019, visitors aged 18 years or older who were able to understand one of the study languages (Dutch, English, French, Spanish, Arabic and Portuguese) were invited to participate.

Prior to June 20, 2019, we excluded patients originating from countries within the European Union (EU) and/or European Economic Area (EEA) who did not possess a citizen service number (CSN), since treatment could not be reimbursed by the central administration office (CAK)-regulation during that time. The CAK is a public service provider that carries out regulations and translates legislations on behalf of the government. CAK-regulation reimburses medical treatment for uninsurable individuals under specific circumstances. As of June 20, 2019, this reimbursement regulation was extended to include individuals originating from countries within the EU or EEA without a CSN and thus after this date, these patients were also invited to participate.

In this report, we provide results on the MRSA screening component of the study. Therefore, only participants with an MRSA screening result were included in the analysis.

### Study procedures

After GP consultation, patients were invited to participate in the study. Eligible patients were provided with study information and if willing to participate, gave oral informed consent. Patients who declined to participate were asked to complete a short questionnaire on demographics and reason(s) for non-participation. All participants were offered an incentive (a ticket for public transportation, socks, toothbrush, shampoo or disinfecting hand gel).

Participants completed two questionnaires: the first filled out together with a research associate (including only information on risk factors for HBV/HCV infection to determine eligibility for HBV/HCV screening) and the second self-administered (including all other information). Information obtained from the questionnaires included sociodemographic variables (age, sex, country of birth, educational level), migration history (year of leaving country of origin, year of arrival in the Netherlands, way of entering the Netherlands, housing situation, the number of housemates they currently live with), antibiotic use (current use and use in the past 6 m) and other variables on potential risk factors (whether or not they had been abroad for more than 24 h in the past 6 m, whether they have ever been admitted or treated in a foreign hospital, had surgery abroad, had a blood transfusion, had paid or had been paid for sex, and injected drugs).

On the day of informed consent, a nasal swab was taken by a research assistant to be screened for MRSA. All positive MRSA diagnoses were added to the electronic health record dossier (EHR) of Kruispost participants to inform healthcare providers in the event of (future) referral to secondary care. Since treatment of MRSA carriage is not indicated outside of hospital settings, we decided not to inform patients of their MRSA status. Participants were informed, however, that their MRSA status would be added to their EHR in case of a positive test.

### Laboratory detection

Collected e-swabs (Copan, Brescia, Italy) were sent to the laboratory of the Public Health Service of Amsterdam by mail at the end of the day of sample collection. Transport time was 24–48 h by mail. The detection of MRSA was done according to the NVMM (Dutch Society for Medical Microbiology) guidelines for laboratory detection of highly-resistant microorganisms [24]. In brief, culture for MRSA was done by overnight enrichment in broth containing 6% NaCl, followed by subculture on selective chromogenic plates (CHROMID MRSA, Biomerieux, Marcy-l’Étoile, France), which were read after 24 and 48 h. All cultures were done at 36 °C. *S. aureus* strains were identified by Maldi-TOF MS (Bruker, Massachusetts, United States of America). MRSA phenotype was confirmed by oxacillin E-test (BioMerieux, Marcy l’Étoile, France) and a PBP2A agglutination test (Alere, Massachusetts, United States of America). Presence of the *mecA* gene was confirmed at the National Institute of Public Health and the Environment (RIVM) by PCR [25]. Isolates were assessed for the presence of Panton-Valentine leukocidin (PVL) gene by PCR [26], which is mainly observed in community-associated MRSA [27] and, in general, is a virulence marker associated with more severe skin and soft tissue infections. Typing of strains isolated in this study was done by Multi-Locus Variable Number Tandem Repeat Analysis (MLVA) as is done with all MRSA strains isolated in the Netherlands in the nationwide MRSA surveillance [28].

### Statistical analyses

Sociodemographics, questions on foreign treatments and antibiotic use were presented by MRSA status. Years since leaving the country of origin and years since arrival in the Netherlands were calculated. Comparisons between groups were made using Fisher exact test for categorical data and by Mann-Whitney U test for continuous data. Prevalence of MRSA carriage and its corresponding Clopper-Pearson 95% confidence interval (CI) were calculated. Odds ratios (OR) comparing odds for MRSA carriage across levels of determinants, along with their 95%CI, were assessed using univariable logistic regression with Firth’s correction. The small number of MRSA-positive samples in our study precluded any multivariable analysis. The significance level was set at *p* < 0.05. All analyses were conducted with Stata 15.1 (StataCorp., College Station, Texas, USA).

## Results

### Participants

**Fig. 1:**
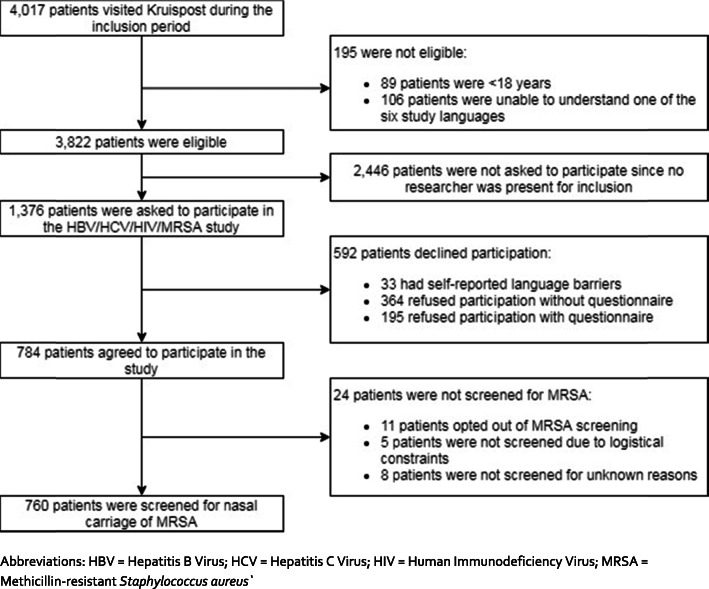
Recruitment strategy of MRSA screening offered to undocumented migrants attending Kruispost in Amsterdam, the Netherlands (*N* = 4017). Abbreviations: HBV = Hepatitis B Virus; HCV = Hepatitis C Virus; HIV = Human Immunodeficiency Virus; MRSA = Methicillin-resistant Staphylococcus aureus`

In total, 4017 patients visited Kruispost during the inclusion period. Of them, 89/4017 (2%) were aged < 18 years and 106/4017 (3%) were unable to understand one of the six study languages. In total, 3822/4017 (95%) eligible patients remained. Of them, 1376 (36%) were invited to participate, and 760 (19.9%) were screened for nasal MRSA carriage. (Fig. 1). Supplementary Table 1 compares the characteristics of those who did versus those who did not participate (restricted to non-participants completing the short questionnaire on demographics). Participants more often originated from Africa and Asia and left their country of origin less recently than non-participants. Furthermore, non-participants were more often European citizens compared to study participants. Supplementary Table 2 shows the reasons for non-participation among patients who completed a short questionnaire on demographics (33% of total non-participants).

Of those who participated, the median age was 40 years (interquartile range (IQR) 31–50) and 58% (442/760) were men. 705/760 participants (93%) were undocumented migrants and 55/760 participants (7%) were uninsured legal residents of Amsterdam. Participants originated mainly from Africa (35%; 267/760), Asia (30%; 229/760) and North or South America (30%; 227/760) and the majority completed secondary school (42%; 320/760) or higher education (36%; 274/760). The ways of entering the Netherlands were diverse across participants, but most indicated arriving on a (now expired) tourist, working or student visa (54%; 406/760), being a rejected asylum seeker (18%; 135/760), or illegally crossing borders (16%; 117/760). Five percent of participants (38/760) reported current use of antibiotics and 25% (186/760) being abroad for more than 24 h in the past 6 m. Of participants, 36% (268/760) reported admission to a foreign hospital and 33% (254/760) had surgery abroad (Table 1).

**Table 1:** Characteristics of undocumented migrants and uninsured legal residents (*N* = 760) and determinants of nasal MRSA carriage, in Amsterdam, the Netherlands, October 2018–October 2019 (univariable logistic regression analyses with Firth’s correction)

	Total (***N*** = 760)		MRSA-negative participants (***N*** = 745)		MRSA-positive participants (***N*** = 15)		Univariable associations	
Sociodemographic variables	*n*	%	*n*	%	*N*	%	OR	95%CI
**Sex**								
Male	442	58%	431	58%	11	73%	Ref	
Female	317	42%	313	42%	4	27%	0.54	0.18–1.62
Other	1	0.1%	1	0.1%	0	0%	*	*
**Age in years, median (IQR)**	40	(31–50)	40	(31–50)	35	(32–48)		
**Age, categorized**								
< 35 years	249	33%	243	33%	6	40%	Ref	
35–49 years	302	40%	295	40%	7	47%	0.95	0.33–2.76
50–64 years	183	24%	181	24%	2	13%	0.52	0.12–2.25
≥ 65 years	26	3%	26	3%	0	0%	0.71	0.04–12.90
**Kruispost target population**								
Undocumented migrant	705	93%	691	93%	14	93%	Ref	
Uninsured legal resident	55	7%	54	7%	1	7%	1.31	0.24–7.21
**Region of birth**								
Europe	36	5%	36	5%	0	0%	Ref	
Asia	229	30%	222	30%	7	47%	2.46	0.14–44.01
Africa	267	35%	261	35%	6	40%	1.81	0.10–32.89
North/South America	227	30%	225	30%	2	13%	0.81	0.04–17.20
**Educational level**								
No school	33	4%	33	4%	0	0%	Ref	
Primary school	129	17%	125	17%	4	27%	2.40	0.13–45.74
Secondary school	320	42%	314	42%	6	40%	1.38	0.08–25.13
Higher education	274	36%	269	36%	5	33%	1.37	0.07–25.28
**Migration history**								
**Year of leaving country of origin, median (IQR)**	2011	(2003–2016)	2011	(2003–2016)	2016	(2009–2016)		
**Year of leaving country of origin, categorized**								
< 2010	335	45%	330	45%	5	33%	Ref	
2010–2017	329	44%	321	44%	8	53%	1.59	0.54–4.69
≥ 2018	88	12%	86	12%	2	13%	1.74	0.38–7.89
**Years since leaving country of origin, median (IQR)**	8	(3–16)	8	(3–16)	3	(3–10)	0.98	0.93–1.04
**Year of arrival in the Netherlands, median (IQR)**	2013	(2006–2017)	2013	(2006–2017)	2016	(2012–2018)		
**Year of arrival in the Netherlands**								
< 2010	279	37%	276	37%	3	20%	Ref	
2010–2017	308	41%	300	41%	8	53%	2.23	0.64–7.84
≥ 2018	167	22%	163	22%	4	27%	2.17	0.53–8.91
**Years since arrival in the Netherlands, median (IQR)**	5	(2–13)	6	(2–13)	3	(1–7)	0.97	0.91–1.04
**Way of entering the Netherlands**								
Expired tourist/working/student visa	406	54%	398	54%	8	53%	Ref	
Rejected asylum seeker	135	18%	133	18%	2	13%	0.88	0.21–3.65
EU citizen	40	5%	39	5%	1	7%	1.78	0.30–10.41
Illegally crossing borders	117	16%	113	15%	4	27%	1.86	0.58–5.94
Legally/other visa**/work	37	5%	37	5%	0	0%	0.63	0.04–11.04
Other/unknown	18	2%	18	2%	0	0%	1.27	0.07–22.80
**Housing situation (multiple answers possible)**								
Lives in BBB facility	56	7%	55	7%	1	7%	1.29	0.23–7.07
Lives with friends/family	401	53%	394	53%	7	47%	0.79	0.29–2.12
Lives in illegal rent	169	22%	166	22%	3	20%	0.97	0.29–3.23
Lives in housing provided by charity	64	9%	63	8%	1	7%	1.11	0.20–6.09
Lives on the streets	60	8%	59	8%	1	7%	1.19	0.22–6.55
Lives in other housing#	51	7%	49	7%	2	13%	2.61	0.66–10.36
**Number of housemates**								
No housemates	105	14%	103	14%	2	13%	Ref	
< 3	335	45%	329	45%	6	40%	0.82	0.19–3.57
3–5	228	31%	223	30%	5	33%	1.02	0.22–4.63
≥ 6	79	11%	77	11%	2	13%	1.34	0.23–7.90
**Antibiotic use**								
**Current antibiotic use**	38	5%	36	5%	2	13%	3.55	0.88–14.25
**Recent antibiotic use**								
Current or < 3 months ago	89	12%	87	12%	2	14%	Ref	
3–6 months ago	54	7%	53	7%	1	7%	0.98	0.13–7.64
> 6 months ago or never used antibiotics	454	63%	447	63%	7	50%	0.59	0.14–2.50
Does not remember when last using antibiotics	125	17%	121	17%	4	29%	1.30	0.27–6.23
**Other potential risk factors**								
**Has been abroad for > 24 h in the past 6 months**	186	25%	183	25%	3	20%	0.85	0.26–2.82
**Ever admitted/treated in a foreign hospital**	268	36%	265	36%	3	20%	0.50	0.15–1.65
**Ever had surgery abroad**	254	33%	250	34%	4	27%	0.77	0.26–2.33
**Ever received a blood transfusion**								
No/unknown	717	94%	703	94%	14	93%	Ref	
Yes	43	6%	42	6%	1	7%	1.71	0.31–9.46
**Ever (been) paid for sex**								
No, never	613	81%	600	81%	13	87%	Ref	
Yes, ever paid	123	16%	121	16%	2	13%	0.92	0.23–3.58
Yes, ever been paid	24	3%	24	3%	0	0%	0.91	0.05–15.72
**Ever injected drugs**	16	2%	15	2%	1	7%	4.88	0.84–28.19

*Insufficient estimation of OR and variance due to ‘0’ count cell

**Includes family visa and Schengen visa

#Includes legal rent, housing for asylum seekers, boats, employers, hotels, crisis care, winter care, campers and forest huts

Missing data: region of birth, *n* = 1; education, *n* = 4; year of leaving country of origin, *n* = 8; year of arrival in the Netherlands, *n* = 6; way of entering the Netherlands, *n* = 7; number of housemates, *n* = 13; current antibiotic use, *n* = 10; recent antibiotic use, *n* = 38; abroad in past 6 m, *n* = 4; admitted to foreign hospital, *n* = 7

*Abbreviations*: *MRSA* methicillin-resistant *Staphylococcus aureus*, *OR* odds ratio, *CI* confidence interval, *IQR* inter quartile range, *EU* European Union, *BBB* bed bath bread

### Prevalence of and risk factors for nasal MRSA carriage

A total of 15 participants were MRSA-positive for nasal carriage, resulting in an overall prevalence of 2.0% (95%CI 1.1 to 3.2%). This prevalence was comparable between undocumented migrants (14/705) (2.0, 95%CI 1.1 to 3.3%) and uninsured legal residents (1/55) (1.8, 95%CI 0.1 to 9.7%). The median age of MRSA carriers was 35 years (IQR 32–48) and 73% (11/15) of carriers was male. Table 1 shows the characteristics of MRSA carriers and non-carriers. As shown in Table 1, MRSA carriage was not associated with any sociodemographic variable, migration history or other possible risk factors.

### MRSA genotyping

Of the 15 isolates from MRSA-positive participants (Table 2), 3 (3/15; 20%) were Panton-Valentine leukocidin (PVL)-positive. Fourteen different MLVA-types were detected. None of the participants had livestock-associated MRSA. Eleven patients (11/15; 73%) had MLVA-types that have never or rarely been found in both the Amsterdam region and nationwide. Four (4/15; 27%) participants had MLVA-types that have been regularly (more than 25 times) isolated in other persons outside the Amsterdam region in the Netherlands within one year before participant MRSA strains were isolated. MLVA cluster analysis of the 15 strains obtained in the present study and other strains isolated in the laboratory of the Public Health Service of Amsterdam showed no genetic relationship between strains from participants with one exception (strains 5 and 13, Table 2). A few strains were included in larger clusters consisting of other, previously isolated strains.

**Table 2:** Genetic characteristics of MRSA isolates of positive participants (*N* = 15)

MRSA-positive participants (***N*** = 15)						
Strain number	MLVA type	MLVA complex	PVL	Residing in the Netherlands since	Number of times MLVA type was diagnosed in the Amsterdam region within one year of date of isolation*	Number of times MLVA type was diagnosed in the Netherlands within one year of date of isolation*
1	MT2502	NC	positive	1995	1	1
2	MT0121	MC0005	negative	2018	1	1
3	MT4112	MC0088	negative	2018	1	1
4	MT6237	MC0008-NC	negative	2016	1	1
5	MT0602	MC0005	negative	2016	8	27
6	MT0486	MC0022	negative	2012	1	11
7	MT0489	MC0254	negative	2014	16	71
8	MT2307	MC0005	negative	2018	1	1
9	MT6179	MC1933	negative	2016	1	2
10	MT0491	MC0022	negative	1990	16	114
11	MT2129	MC0282	positive	2018	1	2
12	MT0321	MC0008	negative	2016	6	35
13	MT0602	MC0005	negative	2009	2	2
14	MT0012	MC0005	negative	2016	1	1
15	MT0432	MC0435	positive	2016	1	1

*Date of isolation refers to the isolation of the MRSA strain from the participant included in this study. This number also includes the strain from the participant

*Abbreviations*: *MRSA* methicillin-resistant *Staphylococcus aureus*, *PVL* Panton-Valentine leukocidin, *MLVA* Multiple Loci Variable Number Tandem Repeat Analysis, *MT* MLVA-type, *NC* nearest complex, *MC* MLVA-complex

## Discussion

In this cross-sectional study among patients attending an NGO health care facility for GP consultations in Amsterdam, the Netherlands, we found a prevalence of 2.0% for nasal MRSA carriage among undocumented migrants and uninsured legal residents. Prevalence did not differ between the two groups. Sociodemographic characteristics, migration history and other potential risk factors for MRSA were not associated with MRSA carriage. Three participants harbored PVL-positive isolates.

The prevalence of nasal MRSA carriage among undocumented migrants and uninsured legal residents from Amsterdam was higher than that reported for the general Dutch population(< 1%) [5, 8, 9]. This finding may partly reflect the prevalence of MRSA carriage in the participants’ country of origin or in countries through which they travelled in transit to the Netherlands. Another possibility is that MRSA was transmitted between undocumented migrants and uninsured legal residents during their stay in the Netherlands. The fact that 14 different MLVA types were found in the MRSA-positive participants would argue for the former hypothesis. Nevertheless, some MLVA types were also frequently identified in other isolates from inhabitants of the Netherlands (i.e. MT0602, MT0489, MT0491 and MT0321). In addition, we did find other MLVA types (MT0121, MT6237, MT2307 and MT0012) that had not been isolated in other persons belonging to the well-known, worldwide occurring MLVA-complexes MC0005 and MC0008. Nevertheless, MLVA types could represent subtle differences from the MLVA-complexes frequently occurring in the Netherlands and might not be recognizably different. It is unknown from routine surveillance data whether these MLVA types are specifically found in migrants.

A meta-analysis on antimicrobial resistance among migrants in Europe found a pooled 8% prevalence of MRSA carriage [11]. A previous retrospective study analyzing screening cultures from asylum seekers who recently arrived in the Netherlands similarly observed a 6% prevalence of nasal MRSA carriage [10]. This prevalence would be almost threefold higher compared to that found in our study. Several hypotheses could explain the varying prevalence of MRSA carriage across studies. There could be differences between asylum seekers and undocumented migrants or uninsured legal residents with respect to housing conditions, country of origin or socioeconomic status. Asylum seekers legally entering the Netherlands, as a result of applying for asylum through the centralized application system, are typically accommodated in an asylum center pending their application. Apart from other (indirect) transmission routes, MRSA is known to spread through skin-to-skin contact in places where crowding and contact occur, such as in schools, camps, gyms, prisons, and possibly asylum centers [29]. Alternatively, MRSA could spread during crowded travel to Europe, such as on refugee boats or in tent camps. Nonetheless, a previous report observed 56 different MLVA types among 104 strains harbored among asylum seekers and considering the wide distribution of countries of origin in their study, the presence of MRSA would be more linked to migrants’ geographical origin than transmission between asylum seekers [10].

It should be noted that the prevalence of nasal MRSA carriage among rejected asylum seekers in our study (2/135 = 1.5, 95%CI 0.2 to 5.3%) was lower than the prevalence of nasal MRSA carriage found in a previous Dutch study of asylum seekers (5.6%) [10]. Asylum seekers whose applications for asylum have been rejected are probably more likely to have been in the Netherlands longer than those currently seeking asylum. Although the median duration needed to clear MRSA carriage is not well known [30–32], MRSA acquired from their country of origin may have cleared spontaneously in our study population by the time they were screened. Another study has demonstrated that asylum seekers living in the Netherlands for more than 1 y had a lower prevalence of MRSA carriage than recently arrived migrants, thereby providing further evidence for this claim [33]. Yet, at a 5.1% prevalence in these longer stay migrants, MRSA carriage would still be higher than found in our study or in the general Dutch population.

We did not find any statistically significant risk factors for nasal MRSA carriage, although we did find that individuals with current antibiotic use or ever injecting drug use tended to have a higher prevalence of MRSA carriage. Antibiotic use [34] and injecting drug use [35] are known risk factors for MRSA, in addition to, among others, recent admittance to or treatment in a foreign hospital and working with livestock [6]. We were unable to confirm these latter findings, mainly owing to the lack of power in our study.

In the Netherlands, the MRSA search and destroy policy ensures that high-risk groups for MRSA are actively screened and pre-emptively isolated upon hospital admission [6]. In 2015, the working group on infection prevention (WIP) additionally advised screening individuals who lived in an asylum center in the previous 2 m for MRSA carriage upon hospital admission [36]. The relatively low MRSA prevalence of 2.0% found in our study compared to the prevalences found in acknowledged high-risk groups for carriage would suggest that screening undocumented migrants and uninsured legal residents admitted to the hospital would be unjustified. However, notwithstanding the small sample size and limited power to identify significant determinants, studies are needed to confirm our findings.

The main strength of our study is that we included diverse populations that have not yet been considered in previous studies. We were able to reach many, generally hard-to-reach, undocumented migrants and uninsured legal residents of Amsterdam and as the study was conducted in six different languages, a broader geographical range of migrants’ country of origin could be included.

However, some limitations need to be addressed. First, as patients were required to have understood one of the six study languages and were taking part in the study with additional HBV, HCV, and HIV screening, the study population was restricted to a convenience sample. Furthermore, Kruispost is a charity-based organization and in order to reduce study costs, we deliberately chose not to have a research associate present for inclusion at all times. The non-random, selective dates of inclusions could have contributed to a lower response. Both the convenience sample and low response might introduce selection bias, which could limit the generalizability of not only all patients at Kruispost, but also of the entire population of undocumented migrants and uninsured legal residents in Amsterdam. Opt-out options were available for MRSA, HBV, HCV and HIV screening; thus the screening for any specific infection was unlikely to influence the attractiveness of this study and reduce the response. Second, over 40 % of patients declined participation and only a small proportion of non-responders completed the short questionnaire on reasons for non-participation. Therefore, it is questionable whether the latter proportion is representative of all non-responders. Moreover, based on this small proportion of non-responders, non-response might be selective. It is unknown to what extent selective non-response and its representativeness for non-responders would have biased our results. Third, more recent migrants were less likely to participate in the study. Since MRSA carriage can spontaneously clear, the prevalence found in our study might be an underestimation compared to that from a study including more recent migrants with potentially more recent exposure to MRSA from their home country. Fourth, it was possible that the pattern of missing data was non-monotonic, potentially biasing our results. Most missing data were observed with respect to recent antibiotic use, but we do not know whether missingness was associated with recent antibiotic use. Fifth, we were unable to reach our target of 1000 participants, causing a lower absolute number of participants with MRSA carriage. Therefore, our study has limited power to evaluate determinants of MRSA carriage. Sixth, since we only assessed nasal MRSA carriage, it is possible that patients carrying MRSA in other locations were missed. Patients with current antibiotic use may have also had false-negative results [37]. These factors could have resulted in an underestimation of the true prevalence of MRSA.

## Conclusion

To the best of our knowledge, this is the first study to examine nasal MRSA carriage among undocumented migrants and uninsured legal residents. Identifying groups with an increased risk of MRSA carriage could lessen the public health consequences of antimicrobial-resistant microorganisms in an interconnected world. Bearing the limited study sample in mind, we show that even though our study population has a higher MRSA prevalence than the general Dutch population, the prevalence is lower than that found in many other studies among migrants and asylum seekers. Future studies should confirm the relatively low prevalence of MRSA carriage among undocumented migrants and uninsured legal residents and may explore explanations for differences between this population and asylum seekers.

## Supplementary information

**Additional file 1: Supplementary Table 1.** Characteristics of included participants (*N* = 784) versus patients who refused participation (but completed a short questionnaire on basic characteristics, *N* = 195) in Amsterdam, the Netherlands, October 2018–October 2019

**Additional file 2: Supplementary Table 2.** Reasons for non-participation among patients who completed a short questionnaire on basic characteristics (*N* = 195) in Amsterdam, the Netherlands, October 2018–October 2019

## Data Availability

The data used and/or analyzed during the current study are available from the corresponding author on reasonable request.

## Abbreviations

CAK: Central Administration Office
CSN: Citizen Service Number
EEA: European Economic Area
EHR: Electronic Health Record
EU: European Union
GP: General practitioner
HBV: Hepatitis B Virus
HCV: Hepatitis C Virus
HIV: Human Immunodeficiency Virus
IQR: Interquartile Range
MLVA: Multi-Locus Variable Number Tandem Repeat Analysis
MRSA: Methicillin-resistant *Staphylococcus aureus*
NVMM: Dutch Society for Medical Microbiology
PVL: Panton-Valentine leukocidin
TB: Tuberculosis
WMO: Medical Research Involving Human Subjects Act
