# Supplementary material for: Nasal carriage of methicillin-resistant Staphylococcus aureus (MRSA) among undocumented migrants and uninsured legal residents in Amsterdam, the Netherlands: a cross-sectional study
Source: Antimicrob Resist Infect Control. 2020 Jul 29;9:118. doi: 10.1186/s13756-020-00785-8 (PMC7391596; doi:10.1186/s13756-020-00785-8)
Supplement: Supplementary file 1 — Additional file 1: Supplementary Table 1. Characteristics of included participants (N = 784) versus patients who refused participation (but completed a short questionnaire on basic characteristics, N = 195) in Amsterdam, the Netherlands, October 2018–October 2019 [file 13756_2020_785_MOESM1_ESM.docx]

Supplementary table 1. Characteristics of included participants (N=784) versus patients who refused participation (but completed a short questionnaire on basic characteristics, N=195) in Amsterdam, the Netherlands, October 2018 - October 2019

|  | | **Included participants**  **(N=784)** | | **Non-included patients**  **(N=195)** | | **P-value*** |
| --- | --- | --- | --- | --- | --- | --- |
|  | | n | % | n | % |  |
| **Demographics** | |  |  |  |  |  |
| **Sex** | |  |  |  |  | .347 |
| Male | | 457 | 58% | 109 | 56% |  |
| Female | | 326 | 42% | 85 | 44% |  |
| Other | | 1 | 0.1% | 1 | 0.5% |  |
| **Age** | |  |  |  |  | .987 |
| <35 years | | 262 | 33% | 66 | 34% |  |
| 35-49 years | | 309 | 39% | 79 | 41% |  |
| 50-64 years | | 187 | 24% | 44 | 23% |  |
| ≥65 years | | 26 | 3% | 6 | 3% |  |
| **Region of birth** | |  |  |  |  | <.001 |
| Europe | | 40 | 5% | 40 | 21% |  |
| Asia | | 234 | 30% | 54 | 28% |  |
| Africa | | 276 | 35% | 41 | 21% |  |
| North/South America | | 233 | 30% | 60 | 31% |  |
| **Year of leaving country of origin** | |  |  |  |  | .002 |
| <2010 | | 346 | 45% | 67 | 36% |  |
| 2010-2017 | | 335 | 43% | 79 | 42% |  |
| ≥2018 | | 93 | 12% | 41 | 22% |  |
| **Year of arrival in the Netherlands** | |  |  |  |  | .131 |
| <2010 | | 287 | 37% | 61 | 32% |  |
| 2010-2017 | | 316 | 41% | 72 | 38% |  |
| ≥2018 | | 173 | 22% | 55 | 29% |  |
| **Way of entering the Netherlands** | |  |  |  |  | <.001 |
| Expired tourist/working/student visa | | 413 | 53% | 98 | 52% |  |
| Rejected asylum seeker | | 139 | 18% | 23 | 12% |  |
| EU citizen | | 47 | 6% | 35 | 19% |  |
| Other/unknown** | | 176 | 23% | 32 | 17% |  |
| * | Differences in variables by MRSA carriage were assessed using a Fisher exact test for categorical data. | | | | | |
| ** | Includes illegally crossing borders, legally, family visa, Schengen visa and work | | | | | |
