# Supplementary material for: Nasal carriage of methicillin-resistant Staphylococcus aureus (MRSA) among undocumented migrants and uninsured legal residents in Amsterdam, the Netherlands: a cross-sectional study
Source: Antimicrob Resist Infect Control. 2020 Jul 29;9:118. doi: 10.1186/s13756-020-00785-8 (PMC7391596; doi:10.1186/s13756-020-00785-8)
Supplement: Supplementary file 2 — Additional file 2: Supplementary Table 2. Reasons for non-participation among patients who completed a short questionnaire on basic characteristics (N = 195) in Amsterdam, the Netherlands, October 2018–October 2019 [file 13756_2020_785_MOESM2_ESM.docx]

Supplementary table 2. Reasons for non-participation among patients who completed a short questionnaire on basic characteristics (N=195) in Amsterdam, the Netherlands, October 2018 - October 2019

| **Reason** | n | % |
| --- | --- | --- |
| Time constraints | 28 | 14% |
| Refused blood draw | 40 | 21% |
| Afraid of test result | 3 | 2% |
| Unwilling to participate in any form of research | 4 | 2% |
| Afraid of being evicted from the Netherlands | 2 | 1% |
| Not interested | 26 | 13% |
| Experiencing language barriers in study information | 19 | 10% |
| Recently tested for HBV/HCV/HIV | 45 | 23% |
| Other reasons | 22 | 11% |
